# Corneal characteristics of Mongolian population with type 2 diabetic peripheral neuropathy in inner Mongolia, China: an assessment using corneal confocal microscopy

**DOI:** 10.1186/s12886-023-03181-z

**Published:** 2023-11-15

**Authors:** Chi Zhang, Lei Zhu, Xiuwen Liang, Yue Li, Guotong Sun, Ji Hu, Honghong Zhang

**Affiliations:** 1 Department of Endocrinology, The Second Affiliated Hospital of Soochow University, 1055 San-Xiang Road, Suzhou, 215000 China; 2 Department of Endocrinology, Hulunbeir People’s Hospital, Hulunbuir, China; 3 Department of Orthopedics, Hulunbeir People’s Hospital, Hulunbuir, China; 4 Department of Cardiology, Hulunbuir Zhong Meng Hospital, Hulunbuir, China; 5 Department of Ophthalmology, Hulunbeir People’s Hospital, Hulunbuir, China; 6 Department of Cardiology, Shouguang Hospital of T.C.M, Weifang, China

**Keywords:** Corneal nerve parameters, Corneal confocal microscopy, Diabetic peripheral neuropathy, Diagnosis, Mongolia

## Abstract

**Objective:**

To quantify corneal nerve fiber parameters in a Mongolian population with diabetic peripheral neuropathy (DPN) by corneal confocal microscopy.

**Methods:**

This study conducted a comprehensive evaluation of 114 participants from Hulunbuir between January 2020 and December 2021. The participants included healthy controls, Mongolian and Han patients with type 2 diabetes mellitus. Demographic, medical, and laboratory data were collected, and neuropathy was evaluated by confocal corneal microscopy. And compare various parameters between Han and Mongolian were performed using SPSS software.

**Results:**

The average waist circumference of Mongolian diabetic patients was larger than that of Han diabetic patients (*P* < 0.05). The mean HbA1c of Mongolian was 9.30 (8.15, 10.30) %, and that of Han was 8.30 (7.20, 9.40) % (*P* = 0.023). The average values of Corneal Nerve Fiber Density (CNFD), Corneal Nerve Fiber Length (CNFL) and corneal nerve branch density (CNBD) in Mongolian diabetic patients were significantly lower than those in Han diabetic patients (*P* < 0.05). The correlation coefficient between CNFL and age was − 0.368. ROC results show that CNBD has a certain diagnostic value for DPN in Mongolian patients with type 2 diabetes and the optimal cut-off point value is 24.99(no./mm^2^), the sensitivity is 80.0%, and the specificity is 77.8%.

**Conclusion:**

The corneal confocal microscopy could possibly represent a promising adjuvant technique for the early diagnosis and assessment of PDN in Mongolian T2DM patients.

## Introduction

Type 2 diabetes mellitus (T2DM) is a chronic condition associated with severe complications, including retinopathy, cardiovascular issues, and diabetic nephropathy [1]. In China, the prevalence of diabetes continues to rise, affecting approximately 10.4% of adults [2]. Globally, the number of individuals with diabetes is expected to reach 783.2 million by 2045 [3]. Diabetic peripheral neuropathy (DPN), the most common form of diabetic neuropathy, affects nearly 50% of diabetes patients and has a significant impact on their health and quality of life [4].

DPN is typically diagnosed based on signs, symptoms, and neurophysiological tests. However, these methods have limitations, and there is a need for more objective and sensitive diagnostic tools. Electromyography (EMG) and nerve conduction velocity (NCV) are considered gold standard tests, but they are mildly invasive (for EMG) and time-consuming [5]. Skin biopsy, another diagnostic option, is invasive and not widely used due to low patient acceptance [6]. Corneal confocal microscopy (CCM) provides a non-invasive and efficient alternative for diagnosing DPN [7]. By directly observing the corneal sub-basal nerve plexus and quantifying nerve fiber parameters, CCM offers valuable insights into DPN without invasive procedures.

In Inner Mongolia, a region inhabited by the Mongolian population, there is a high incidence of T2DM due to genetic and environmental factors. The unique living environment, dietary habits, and lifestyle of the Mongolian population contribute to their increased risk of T2DM [8–10]. However, it remains uncertain whether the incidence and characteristics of DPN differ between Mongolian and Han populations. Additionally, there is a lack of normative CCM reference values specifically for the Mongolian population.

This study aims to address these gaps by comparing clinical characteristics and CCM parameters between Mongolian and Han patients with T2DM and DPN in Inner Mongolia. The findings will enhance our understanding of DPN in different ethnic groups and contribute to more accurate and efficient diagnosis and management of this complication. Establishing specific diagnostic parameters for CCM in the Mongolian population will provide valuable guidance for diagnosing DPN in Chinese adults.

## Materials and methods

### Research objects

This study evaluated 114 participants at Hulunbuir People’s Hospital in Inner Mongolia from January 2020 to December 2021. The participants included 10 normal healthy controls (5 Mongolian and 5 Han), 33 Mongolian patients with type 2 diabetes mellitus (including 15 patients with DPN and 18 patients without DPN). Additionally, there were 71 Han patients with type 2 diabetes mellitus (including 37 patients with DPN and 34 patients without DPN). Compare the differences in various parameters between healthy individuals, patients with DPN, and patients without DPN among Mongolian and Han ethnic groups. Inclusion criteria: ① patients with type 2 diabetes mellitus. ② Live in Hulunbuir for more than 10 years. ③ All the volunteers participating in the questionnaire can understand the questionnaireaccurately and cooperate with the successful completion of the questionnaire survey. exclusion criteria: ① Volunteers with acute disease, history of chronic gastrointestinal disease, hypertension, cerebrovascular disease, malignant tumor, autoimmune disease (systemic lupus erythematosus, systemic sclerosis, Crohn’s disease, etc.), vitamin B12 or folate deficiency, hypothyroidism, hepatic or renal dysfunction, cervical or lumbar spine disease, and central neurodegenerative diseases (including Parkinson’s disease, multiple sclerosis, Alzheimer’s disease, Huntington’s disease, and dementia). ② Volunteers with a family history of genetic disorders (including but not limited to hereditary neuropathy), history of ocular trauma, diseases, corneal disorders, surgery, contact lens wearing, alcohol abuse, and toxic or chemotherapeutic drug exposure were also excluded. ③ Pregnant or breastfeeding women and vegetarians. This study was approved by the Ethics Committee of Hulunbuir People’s Hospital and conformed to the purposes of the Declaration of Helsinki. Each participant signed an informed consent form before the study.

### Demographic, medical, and laboratory data

Basic demographic characteristics of each participant, such as ethnicity, sex, age, weight, height, waist circumference, history of smoking, alcohol consumption, and duration of type 2 diabetes, were obtained by trained researchers. All participants also received fasting blood glucose, percentage glycosylated hemoglobin (HbA1c), total cholesterol, high-density lipoprotein (HDL) and low-density lipoprotein (LDL) cholesterol, triglycerides, serum uric acid, serum creatinine, alanine aminotransferase, ASpartate aminotransferase, glutamyl transpeptidase, and urinary microprotein. All participants underwent detailed evaluation for neuropathy and CCM.

### Diagnosis and evaluation of DPN

The diagnosis of diabetic peripheral neuropathy (DPN) was conducted through nerve conduction velocity (NCV) examination. All participants underwent NCV testing using the Medoc Keypoint EMG machine. The subjects remained in a supine position at an ambient temperature of 26–28 °C, with limb surface temperature maintained between 34 and 36 °C in a quiet environment free from interference. The testing was carried out by trained professionals. The nerves assessed for each patient included the sural and tibial nerves. The electrophysiological parameters measured encompassed sensory and motor nerve conduction velocities. Sensory nerve conduction was determined using surface skin electrodes in an orthodromic fashion, while motor nerve conduction employed disk-like surface electrodes to record muscle action potentials. Stimulation intensity was adjusted to elicit compound muscle action potentials or sensory nerve action potentials. Measurements of the maximum amplitude of the waveform were repeated several times until there was no further variation in waveform or amplitude. The presence of 2 or more slowing items is positive (cut-off value used for the NCV was 42.3 m/s), and is judged as DPN. Nerve conduction velocity data are shown in Table 1.

**Table 1:** Nerve conduction velocity data

	Motor conduction velocities of the tibial nerve	Sensory *conduction* velocities of the sural nerve
Han patients with DPN	45.91 ± 2.98	47.51 ± 3.85
Han patients without DPN	50.71 ± 3.95	49.03 ± 5.01
Mongolian patients with DPN	44.3 ± 2.31	44.48 ± 3.19
Mongolian patients without DPN	48.68 ± 5.10	47.26 ± 3.66
Han healthy controls	48.76 ± 1.11	54.96 ± 1.93
Mongolian healthy controls	52.88 ± 2.61	52.14 ± 9.94

### Confocal corneal microscopy

The Heidelberg Third-generation retinal laser tomography system (HRT 3) and Rostock corneal module Assembly (RCM) were used to capture images of each participant’s corneal sub-basal nerve plexus. These procedures were performed by a skilled and experienced ophthalmologist, taking into account depth, focal position, and contrast [11, 12]. Five images with high definition and reflecting the corneal sub-basal nerve plexus were selected for analysis to assess the following parameters: Corneal Nerve Fiber Length (CNFL, mm/mm2), which represents the total length of nerve fibers per square millimeter; Corneal Nerve Fiber Density (CNFD, no./mm2), which indicates the number of main nerve fibers per square millimeter; and Corneal Nerve Branch Density (CNBD, no./mm2), which measures the number of branches from the main nerve per square millimeter. These parameters (CNFL, CNFD, and CNBD) were evaluated using ACC Metrics, a fully automated analysis software provided by the University of Manchester, UK.

### Statistical analysis

SPSS 25.0 software was used for data analysis. All measurement data were tested for normality by Shapiro-Wilk method, and corresponding statistics and *P* values were obtained. When *P* > 0.05 indicates a normal distribution. The measurement data in accordance with normal distribution were expressed as mean ± standard deviation, and those not in accordance with normal distribution were expressed as median (quartile). The variables with normal distribution and homogeneity of variance were analyzed by two independent sample t-test, and its statistic was t value. The corrected T-value was used for uneven variance. When the distribution is not normal, the rank sum test is used, and its statistic is the z-value. The counting data were expressed as percentage (%), processed by Chi-square test or Fisher’ Exact Test. *P* values < 0.05 was considered significant and the difference was statistically significant. Potential correlations were assessed using the Spearman rank correlation analysis or Pearson correlation coefficient. Receiver operating characteristic (ROC) analysis was performed to generate ROC curves. The area under the curve (AUC) for each index was compared, and the optimal cut-off point value was determined by maximizing Youden’s index.

## Results

### Parameters of corneal nerve fibers and demographic characteristics in Mongolian and Han patients

The results showed that the mean waist circumference of Mongolian was 99.00 (91.50,102.00) cm, and that of Han was 93.00 (87.00,98.00) cm, and there was a statistical difference between the two groups (*P* = 0.014). The mean HbA1c of Mongolian was 9.30 (8.15, 10.30) %, and that of Han was 8.30 (7.20, 9.40) %, and there was a significant difference between the two groups (*P* = 0.023). There were no significant differences in liver function, lipid metabolism, renal function, uric acid and fasting blood glucose. The mean values of CNFD in type 2 diabetic patients in Mongolian group and Han group were 18.74 (16.24,23.74) and 24.99 (19.99,28.74) no./mm^2^, respectively, and there was significant statistical difference between the two groups (*P* = 0.001). The mean values of CNFL in type 2 diabetic patients in Mongolian group and Han group were 13.81 ± 3.15 and 15.39 ± 3.47 mm/mm^2^, respectively, and there was a significant difference between the two groups (*P* = 0.029). The mean values of CNBD in type 2 diabetic patients in Mongolian group and Han group were 25.00(16.24,41.56) and 33.74(18.75,51.24) no./mm^2^, respectively, and there was no significant difference between the two groups (*P* = 0.075). See Table 2 for details.

**Table 2:** Comparison of corneal nerve fiber parameters and baseline data between Mongolian and Han patients with type 2 diabetes mellitus

Characteristics	Mongolian (*n* = 33)	Han (*n* = 71)	Statistical value	*P* value
Diabetes duration (month)	120.00 (60.00,210.00)	84.00 (30.00,168.00)	-1.248^b^	0.212
Gender (male/female)	23/10	46/25	0.243^c^	0.622
Age (years)	55.00 (46.00,63.50)	57.00 (51.00,61.00)	-0.337^b^	0.706
Smoke (yes/no)	13/20	26/45	0.074^c^	0.786
Drink (yes/no)	16/17	34/37	0.003^c^	0.955
Waist circumference (cm)	99.00 (91.50,102.00)	93.00 (87.00,98.00)	-2.458^b^	0.014
BMI (kg/m^2^)	26.90 (24,85,28.90)	26.10 (24,50,27.90)	-0.940^b^	0.347
MALB (mg/L)	10.70 (3.10,57.00)	6.90 (3.20,18.60)	-0.842^b^	0.400
HbA1c (%)	9.30 (8.15, 10.30)	8.30 (7.20,9.40)	-2.271^b^	0.023
ALT (U/L)	21.00 (15.00,32.50)	21.00 (14.00,32.00)	-0.304^b^	0.761
AST (U/L)	20.00 (16.00,23.00)	19.00 (15.00,24.00)	-0.420^b^	0.675
GGT (U/L)	27.00 (21.00,45.50)	30.00 (20.00,47.00)	-0.269^b^	0.788
FPG (mmol/L)	8.86 ± 3.35	7.81 ± 2.55	-1.601^a^	0.116
CREA (umol/L)	61.50 (55.00,70.95)	61.40 (53.30,73.20)	-0.014^b^	0.989
UA (umol/L)	333.36 ± 79.97	360.30 ± 102.22	1.334^a^	0.185
CHOL (mmol/L)	4.45 (3.78,5.64)	4.60 (3.82,5.45)	-0.129^b^	0.897
TG (mmol/L)	1.80 (1.22,2.52)	1.76 (1.20,2.40)	-0.402^b^	0.688
HDL-C (mmol/L)	1.03 (0.90,1.20)	0.98 (0.86,1.12)	-1.125^b^	0.261
LDL-C (mmol/L)	2.82 (1.82,3.49)	2.75 (1.99,3.43)	-0.112^b^	0.911
CNFD (no./mm^2^)	18.74 (16.24,23.74)	24.99 (19.99,28.74)	-3.340^b^	0.001
CNBD (no./mm^2^)	25.00 (16.24,41.56)	33.74 (18.75,51.24)	-1.778^b^	0.075
CNFL (mm/mm^2^)	13.81 ± 3.15	15.39 ± 3.47	2.215^a^	0.029

*BMI *Body mass index, *MALB *Urinary microprotein, *HbA1c *Percentage of glycosylated hemoglobin, *ALT *Alanine aminotransferase, *AST *Aspartate aminotransferase, *GGT *Glutamyl transpeptidase, *FPG *Fasting blood glucose, *CREA *Blood creatinine, *UA *Blood uric acid, *CHOL *Total cholesterol, *TG *Triglyceride, *HDL-C *High density lipoprotein cholesterol, *LDL-C *Low-density lipoprotein cholesterol, *CNFD *Corneal nerve fiber density, *CNBD *Corneal nerve branch density, *CNFL *Corneal nerve fiber length

^a^t value

^b^Z value

^c^X^2^ values. *P* < 0.05 indicates statistical significance

### Comparison of patients with non-diabetic peripheral neuropathy between Mongolian population and Han population

Among 33 Mongolian patients with type II diabetes, 18 patients without DPN. Among 71 Han patients with type II diabetes, 34 patients without DPN. The results showed that the mean duration of disease in Mongolian patients without DPN was 102.00 (24.00,156.00) months, and that in Han patients without DPN was 36.00 (7.50,96.00) months, and the difference was statistically significant (*P* = 0.034). The mean waist circumference of Mongolian was 100.00 (94.50,107.25) cm, and that of Han was 92.50 (86.75,96.50) cm, and there was a statistical difference between the two groups (*P* = 0.002). There were no significant differences in HbA1c, liver function, lipid metabolism, renal function, uric acid and fasting blood glucose. The mean values of CNFD in Mongolian patients without DPN and Han patients without DPN were 19.98 ± 4.65 and 23.56 ± 6.35 no./mm^2^, respectively, with significant statistical difference between the two groups (*P* = 0.040). There was no significant difference in CNBD (*P* = 0.532) and CNFL (*P* = 0.183) between the two groups. See Table 3 for details.

**Table 3:** Comparison of patients with Non-diabetic peripheral neuropathy between Mongolian population and Han population

	Mongolian with NDPN(*n* = 18)	Han with NDPN (*n* = 34)	Statistical value	*P* value
Diabetes duration (month)	102.00 (24.00,156.00)	36.00 (7.50,96.00)	-2.120^b^	0.034
Gender (male/female)	15/3	20/14	3.213^c^	0.073
Age (years)	49.06 ± 12.27	53.27 ± 10.52	1.296^a^	0.201
Smoke (yes/no)	7/11	10/24	0.480^c^	0.488
Drink (yes/no)	13/5	12/22	6.429^c^	0.011
Waist circumference (cm)	100.00 (94.50,107.25)	92.50 (86.75,96.50)	-3.081^b^	0.002
BMI (kg/m^2^)	28.40 (25.15,29.63)	26.10 (24.85,28.45)	-1.270^b^	0.204
MALB (mg/L)	6.85 (2.70,112.28)	5.95 (2.90,43.00)	-0.346^b^	0.729
HbA1c (%)	9.10 (8.08,10.20)	8.60 (7.35,9.70)	-0.972^b^	0.331
ALT (U/L)	22.00 (14.25,37.75)	24.50 (14.75,36.00)	-0.144^b^	0.885
AST (U/L)	21.00 (15.50,29.25)	21.00 (16.75,27.00)	-0.106^b^	0.916
GGT (U/L)	29.50 (24.00,53.25)	39.00 (21.50,65.75)	-0.462^b^	0.644
FPG (mmol/L)	8.30 (7.20,10.75)	8.04 (6.24,9.56)	-1.222^b^	0.222
CREA (umol/L)	62.43 ± 13.99	62.18 ± 15.51	-0.056^a^	0.956
UA (umol/L)	350.50 (307.75,412.00)	354.50 (305.00,451.00)	-0.875^b^	0.381
CHOL (mmol/L)	4.64 (3.75,5.86)	4.69 (4.02,5.82)	-0.346^b^	0.729
TG (mmol/L)	1.65 (1.18,2.25)	1.91 (1.45,2.75)	-0.924^b^	0.356
HDL-C (mmol/L)	1.01 (0.89,1.10)	0.99 (0.84,1.12)	-0.318^b^	0.751
LDL-C (mmol/L)	2.94 (2.18,3.)	2.76 (2.24,3.44)	-0.029^b^	0.977
CNFD (no./mm^2^)	19.98 ± 4.65	23.56 ± 6.35	2.108^a^	0.040
CNBD (no./mm^2^)	33.12 (23.43,42.96)	36.24 (22.49,51.87)	-0.625^b^	0.532
CNFL (mm/mm^2^)	14.48 ± 3.08	15.74 ± 3.26	1.352^a^	0.183

^a^t value

^b^Z value

^c^X^2^ values

### Comparison of patients with diabetic peripheral neuropathy between Mongolian population and Han population

The results showed that the mean HbA1c of Mongolian patients with DPN was 9.30 (8.20,10.70) %, and the mean HbA1c of Han patients with DPN was 7.90 (7.05,8.90) %, and there was a statistical difference between the two groups (*P* = 0.024). There were no significant differences in liver function, lipid metabolism, renal function, uric acid and fasting blood glucose. The mean values of CNFD in Mongolian patients with DPN and Han patients with DPN were 18.91 ± 5.78 and 23.78 ± 7.84 no./mm^2^, respectively, and there was a significant difference between the two groups (*P* = 0.035). CNBD (*P* = 0.036), CNFL (*P* = 0.064), there was significant difference between the two groups. See Table 4 for details.

**Table 4:** Comparison of patients with diabetic peripheral neuropathy between Mongolian population and Han population

	Mongolian with DPN (*n* = 15)	Han with DPN (*n* = 37)	Statistical value	*P* value
Diabetes duration (month)	150.73 ± 94.24	148.43 ± 91.00	-0.082^a^	0.935
Gender (male/female)	8/7	26/11	1.353^c^	0.245
Age (years)	61.00 (54.00,68.00)	58.00 (54.00,64.00)	-0.920^b^	0.357
Smoke (yes/no)	6/9	16/21	0.046^c^	0.830
Drink (yes/no)	3/12	22/15	6.657^c^	0.010
Waist circumference (cm)	96.00 (88.00,100.00)	93.00 (88.00,99.00)	-0.162^b^	0.871
BMI (kg/m^2^)	25.50 (24.60,27.00)	26.10 (24.30,27.70)	-0.364^b^	0.716
MALB (mg/L)	11.10 (5.90,23.20)	8.00 (3.40,15.95)	-1.050^b^	0.294
HbA1c (%)	9.30 (8.20,10.70)	7.90 (7.05,8.90)	-2.264^b^	0.024
ALT (U/L)	21.00 (15.00,26.00)	18.00 (12.00,26.50)	-0.668^b^	0.504
AST (U/L)	20.00 (16.00,21.00)	17.00 (14.00,22.00)	-0.699^b^	0.485
GGT (U/L)	24.00 (16.00,45.00)	24.00 (19.50,38.00)	-0.374^b^	0.708
FPG (mmol/L)	8.54 ± 3.92	7.47 ± 2.42	-0.988^a^	0.336
CREA (umol/L)	63.00 (48.90,71.00)	65.60 (52.10,73.75)	0.333^b^	0.739
UA (umol/L)	312.93 ± 89.91	346.51 ± 92.68	1.194^a^	0.238
CHOL (mmol/L)	4.45 (3.94,5.42)	4.36 (3.64,5.32)	-0.525^b^	0.599
TG (mmol/L)	2.10 (1.40,3.40)	1.50 (1.10,2.06)	-1.546^b^	0.122
HDL-C (mmol/L)	1.12 (0.91,1.25)	0.98 (0.87,1.14)	-1.192^b^	0.233
CNFD (no./mm^2^)	18.91 ± 5.78	23.78 ± 7.84	2.173^a^	0.035
CNBD (no./mm^2^)	20.00 (13.74,24.99)	30.00 (18.12,49.37)	-2.101^b^	0.036
CNFL (mm/mm^2^)	13.01 ± 3.14	15.06 ± 3.67	1.897^a^	0.064

^a^t value

^b^Z value

^c^X^2^ values

### Comparison of corneal nerve fiber parameters between Mongolian and Han healthy people

As can be seen from Table 5, there is no statistical significance in the differences of CNFD, CNBD and CNFL between healthy Mongolian population and healthy Han population. See Table 5 for details.

**Table 5:** Corneal nerve parameters of Han and Mongolian healthy controls

	Healthy Han population (*n* = 5)	Healthy Mongolian population (*n* = 5)	Statistical value	*P* value
CNFD (no./mm^2^)	23.28 ± 4.29	20.24 ± 4.09	1.148	0.284
CNBD (no./mm^2^)	43.74 (32.49,49.99)	21.24 (19.37,45.62)	-1.358	0.175
CNFL (mm/mm^2^)	16.31 ± 0.98	14.51 ± 2.49	1.502	0.171

### Correlation analysis between corneal nerve fiber parameters and demographic characteristics in Mongolian population

The results of correlation analysis showed that CNFD and CNBD were not significantly correlated with demographic characteristics, with *P* > 0.05. Moreover, the correlation coefficient between CNFL and age was − 0.368, *P* value was 0.035. See Table 6 for details.

**Table 6:** Correlation analysis of corneal nerve fiber parameters in Mongolian population (*n* = 33)

	CNFD		CNBD		CNFL	
	The correlation coefficient	*P* value	The correlation coefficient	*P* value	The correlation coefficient	*P* value
Diabetes duration	-0.071^2)^	0.694	-0.096^2)^	0.596	-0.190^b^	0.289
Age	-0.078^2)^	0.665	-0.312^2)^	0.077	-0.368^b^	0.035
Waist circumference	-0.052^1)^	0.772	0.004^1)^	0.980	0.008^a^	0.967
BMI	-0.030^1)^	0.868	-0.078^1)^	0.666	-0.011^a^	0.950
MALB	-0.184^2)^	0.304	-0.218^2)^	0.224	-0.272^b^	0.126
HbA1c	-0.088^1)^	0.627	0.099^1)^	0.583	-0.014^a^	0.938
ALT	-0.076^2)^	0.675	0.015^2)^	0.934	0.062^b^	0.730
AST	0.012^2)^	0.947	0.261^2)^	0.142	0.206^b^	0.251
GGT	-0.108^2)^	0.550	-0.010^2)^	0.956	0.020^b^	0.914
PFG	-0.286^1)^	0.106	0.065^1)^	0.718	0.030^a^	0.870
CREA	0.069^1)^	0.701	0.202^1)^	0.260	0.155^a^	0.390
UA	0.044^1)^	0.807	0.109^1)^	0.545	0.070^a^	0.698
CHOL	0.040^1)^	0.823	0.039^1)^	0.831	0.185^a^	0.302
TG	-0.267^2)^	0.134	-0.338^2)^	0.055	-0.245^b^	0.154
HDLC	-0.026^2)^	0.888	0.012^2)^	0.947	0.006^b^	0.973
LDLC	0.184^1)^	0.304	0.163^1)^	0.365	0.291^a^	0.101

^a^The r value

^b^The rs values

### The receiver operating characteristic curve of corneal nerve parameters in diagnosis of DPN in Mongolian patients with type II diabetes mellitus

**Fig. 1:**
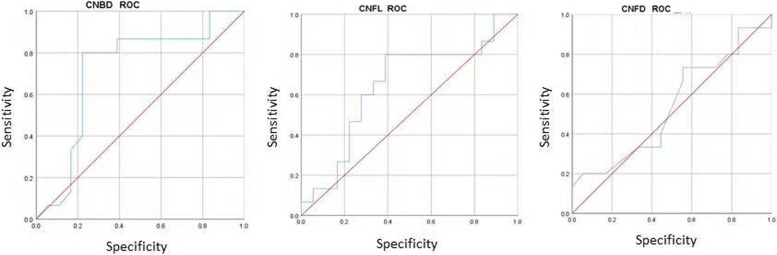
The receiver operating characteristic curve of corneal nerve parameters in diagnosis of DPN

The receiver operating characteristic (ROC) curves of corneal nerve parameters CNFL, CNFD and CNBD were drawn, and the area under the curve (AUC) of each corneal nerve parameter was calculated (Fig. 1; Table 7). The results suggested that corneal nerve parameter CNBD has certain diagnostic value for DPN in Mongolian patients with type 2 diabetes mellitus. According to ROC curve and Youden index maximization, the optimal cut-off point value is 24.99(no./mm^2^), the sensitivity is 80.0%, and the specificity is 77.8%.

**Table 7:** Area under ROC curve comparison of corneal nerve fiber parameters

Corneal nerve parameters	AUC	95% CI	*P* value
CNFD	0.537	0.034–0.740	0.718
CNBD	0.717	0.528–0.906	0.034
CNFL	0.644	0.448–0.841	0.159

## Discussion

Our study showed that compared with Han diabetic patients, Mongolian T2DM patients had larger waist circumference and higher percentage of HbA1c. The corneal nerve fiber parameters (CNFD, CNFL, CNBD) of Mongolian T2DM patients were significantly lower than those of Han diabetic patients. Mongolian and Han diabetic patients were divided into neuropathy group and non-neuropathy group according to EMG status. In the patients without DPN group, compared with Han diabetic patients, Mongolian diabetic patients had larger waist circumference, longer disease duration, and larger corneal nerve fiber parameters than Han diabetic patients. In the DPN group, Mongolian patients had a higher percentage of glycosylated hemoglobin and corneal nerve fiber parameters than Han diabetic patients. The ROC curve showed that the corneal nerve parameter CNBD has a good diagnostic value for DPN in Mongolian type 2 diabetic patients.

Diabetes mellitus is a common endocrine and metabolic disease, mainly manifested by abnormal elevation of blood glucose. Diabetic neuropathy is the most common chronic complication of diabetes, and the initial diagnosis rate is low, most of which cannot be paid enough attention in the initial stage. With the continuous progress of the disease, there will be peripheral nerve dysfunction and other manifestations, which will reduce the quality of life of patients, and may lead to diabetic foot [13], amputation [14] and even death [15]. Since its pathogenesis is still unclear, early diagnosis and early intervention are very important to delay the progression of the disease. Unmyelinated small nerves are the first nerve fibers to be damaged in the course of DPN [16]. Recent studies have found that the density of intradermal small nerve fibers decreases in diabetic patients with normal nerve conduction function, and the decrease of epidermal nerve fiber density can also be detected in patients with abnormal glucose tolerance [17–19]. Due to the increasing attention paid to the morphological changes of small nerve fibers in the early diagnosis of DPN, the sensitivity of nerve conduction measurement to evaluate these nerves is weak [20, 21]. Many scholars at home and abroad have begun to turn their attention to CCM, which can scan the corneal nerves in vivo quickly.

At present, it is clear that type 2 diabetes is caused by the combination of environmental factors and genetic factors, including modern lifestyle, excessive nutrition, insufficient physical activity, chemical toxicants and so on. Due to lifestyle changes away from the city for a long time, Mongolian nomads have their own unique lifestyle and eating habits, and therefore in the process of long-term life formed unique metabolic pathway, so as to adapt to the environment factors. Besides environmental factors, genetic factors may also play a role. We found that the waist circumference of Mongolian patients with type 2 diabetes mellitus was significantly larger than that of Han patients. Studies have found that abdominal obesity is more closely associated with the risk of insulin resistance than systemic obesity [22]. The accumulation of abdominal fat may contribute to insulin resistance by stimulating the formation of metabolites produced by lipids, hormones and cytokines, which in turn increases the risk of metabolic and cardiovascular diseases. Studies have shown that body fat distribution and adipose tissue dysfunction are key factors for obesity-related insulin resistance and metabolic diseases, and the accumulation of adipose tissue in the upper body (abdomen) is associated with obesity-related comorbidities and even all-cause mortality [23]. As Mongolian people are still nomadic, they have a large amount of activity, and the area is extremely cold, so their energy consumption increases. In terms of diet, almost all meat and dairy products are the main, protein and fat intake, and fresh vegetables and fruits less. Therefore, the waist circumference of Mongolian patients is significantly larger than that of Han. A reduction in HbA1c levels is strongly associated with a reduction in complications in patients with diabetes [24].The results from the UK Prospective Diabetes Study (UKPDS) [25] showing that a 1% decrease in HbA1c was associated with a 21% reduction in the risk of all diabetes-related end points and diabetes-related mortality (*P* < 0.01) and 14% lower risk of myocardial infarction (*P* < 0.01)and 37% lower risk of microvascular complications (*P* < 0.01).In our study, we found that the level of HbA1c in Mongolian patients with DPN was significantly higher than that in Han patients, indicating that Mongolian patients had poor blood glucose control, more severe nerve damage, and were more likely to be complicated with diabetic peripheral neuropathy.

A study by Nitoda E et al. [26] showed that nerve fiber alterations of the subbasal nerve plexus of diabetic corneas appear to progress in parallel with diabetic retinopathy and peripheral diabetic neuropathy. From our study, we found that the corneal nerve fiber parameters of Mongolian patients with type 2 diabetes mellitus were significantly lower than those of Han patients. At the same time, the corneal nerve fiber parameters of Mongolian healthy people and Han healthy participants were still low, although there was no significant statistical difference between them, it was considered that the reason might be due to the small sample size. Therefore, we concluded that the corneal nerve fiber parameters of the Mongolian population as a whole were lower than those of the Han population. In the correlation analysis between corneal nerve fiber parameters and baseline data, it was found that corneal nerve fiber parameters and gender, smoking, drinking history, waist circumference, BMI were not correlated with CNFD, CNFL, CNBD. The correlation analysis between the corneal nerve fiber parameters and baseline data also found that CNFL was negatively correlated with age. This result is consistent with the findings of Tavakoli et al. [27], which demonstrated that CNFL gradually decrease with age, while CNBD does not show a significant correlation with age. Furthermore, the three parameters (CNFD, CNFL, and CNBD) do not exhibit a significant correlation with height, weight, and BMI. In our study, we also did not observe a correlation between CNFD and age. Several more recent studies have also reported on the factors associated with changes in corneal nerve parameters. Although not mentioned in this study, these factors include but are not limited to the duration of diabetes, glycemic control, blood pressure, lipid profile, body mass index, and presence of diabetic complications [28]. It is important to consider the findings of these studies in future research to gain a comprehensive understanding of the risk factors contributing to corneal nerve loss.

Corneal confocal microscopy is a novel diagnostic technique for the detection of nerve damage and repair in a range of peripheral neuropathies, in particular diabetic neuropathy. The application of CCM to evaluate the severity of DPN has been recognized by many studies, but there are few studies on differences between populations in different living environments. Our study showed that the corneal nerve parameter CNBD had certain diagnostic value for DPN in Mongolian patients with type 2 diabetes mellitus. According to the ROC curve, the optimal cut-off point value was 24.99(no./mm^2^), the sensitivity was 80.0%, and the specificity was 77.8%. Our research shows that corneal confocal microscopy could possibly represent a promising adjuvant technique for the early diagnosis and assessment of PDN in Mongolian T2DM patients. However, normative reference values are required to enable clinical translation and wider use of this technique.

There are also some limitations in our study. First, the sample size of the healthy population used as the control group was relatively small. Second, vitamin B12 or folate levels were not tested; individuals with autoimmune diseases were only excluded through a detailed questionnaire and physical examination. Third, there is a lack of diagnostic evaluation data such as positive detection rate and negative detection rate. Future prospective multicenter studies are necessary to confirm our results.

## Conclusion

There were differences in demographic characteristics and corneal nerve fiber parameters between Mongolian type 2 diabetic patients and Han type 2 diabetic patients. The corneal nerve fiber parameter CNFL is correlated with age. Corneal nerve parameter CNBD has certain diagnostic value for DPN in Mongolian patients with type 2 diabetes mellitus. The corneal confocal microscopy could possibly represent a promising adjuvant technique for the early diagnosis and assessment of DPN in Mongolian T2DM patients. This study provides a reference for clinical practice and research with this technique.

## Data Availability

All data generated or analyzed during this study are included in this published article.
